# 托珠单抗早期干预细胞因子释放综合征对嵌合抗原受体T细胞治疗的影响

**DOI:** 10.3760/cma.j.issn.0253-2727.2023.12.009

**Published:** 2023-12

**Authors:** 莉莉 周, 世光 叶, 萍 李, 晓晨 唐, 爱斌 梁

**Affiliations:** 同济大学附属同济医院血液科，上海 200065 Department of Hematology, Tongji Hospital, Tongji University School of Medicine, Shanghai 200065, China

**Keywords:** 细胞因子释放综合征, 嵌合抗原受体T细胞, 托珠单抗, Cytokine release syndrome, Chimeric antigen receptor T cell, Tocilizumab

## Abstract

**目的:**

分析早期使用托珠单抗缓解细胞因子释放综合征（CRS）对嵌合抗原受体T细胞（CAR-T细胞）治疗效果的影响。

**方法:**

收集2015 年10月至2021年7月同济大学附属同济医院血液科输注靶向CD19 CAR-T细胞后发生CRS并接受托珠单抗治疗的22例急性淋巴细胞白血病（ALL）患者资料。按照托珠单抗干预的时机分为常规组和早期干预组，患者持续高热4 h即接受托珠单抗治疗的为早期干预组。回顾性分析两组之间的临床资料、CRS分级和无事件生存。

**结果:**

与发生了严重CRS后常规使用托珠单抗相比，早期干预组没有患者因CRS死亡，没有增加神经毒性风险。11例（84.62％）患者获得微小残留病阴性的完全缓解。常规组和早期干预组的中位无事件生存时间分别为2（95％*CI* 0～5）个月、7（95％*CI* 3～11）个月。

**结论:**

早期使用托珠单抗干预患者的CRS有助于减少重症CRS的发生，并为CAR-T细胞治疗ALL产生的CRS反应提供了更优化的治疗策略。

## Abstract (en)

**Objective:**

This study aimed to evaluate the effect of early tocilizumab intervention to relieve cytokine release syndrome（CRS）following chimeric antigen receptor T cell（CAR-T）therapy.

**Methods:**

Twenty-two patients with acute lymphoblastic leukemia who received tocilizumab to relieve CRS response after CAR-T cell infusion in our research center from October 2015 to July 2021 were retrospectively analyzed. According to the timing of tocilizumab intervention, patients were divided into the conventional and early intervention groups. Patients who received tocilizumab treatment after sustained high fever for 4 h were included in the early intervention group. The clinical data, CRS grade, and event-free survival（EFS）between the two groups were evaluated.

**Results:**

Compared with patients who used tocilizumab after severe CRS, no patients in the early intervention group died from CRS, and there was no increased risk of neurotoxicity. Eleven patients（84.62％）achieved complete remission with minimal residual lesions. The median EFS of patients in the early intervention and conventional groups was 2（95％ *CI* 0–5）and 7（95％ *CI* 3–11）months, respectively.

**Conclusion:**

Early tocilizumab intervention in patients with CRS reduces severe CRS and provides a more optimized therapeutic strategy for CRS caused by CAR-T cell therapy.

嵌合抗原受体T细胞（CAR-T细胞）介导的免疫治疗在多项治疗血液系统恶性肿瘤的临床研究中呈现出显著疗效[1]–[3]，尤其是靶向CD19 CAR-T细胞治疗急性淋巴细胞白血病（ALL）和淋巴瘤已经获得了广泛的认可。对于B-ALL患者而言，靶向CD19 CAR-T细胞治疗过程中出现的最常见最严重的免疫治疗相关不良反应是细胞因子释放综合征（CRS）和免疫效应细胞相关神经毒性综合征（ICANS）。CRS一般出现在CAR-T细胞输注后的两周内，可累及全身各个脏器，引起的症状类型广泛，轻重不一。严重CRS可发生重要脏器功能衰竭而危及患者生命。

IL-6是介导炎症反应的主要细胞因子，也是一种驱动CRS的细胞因子。托珠单抗是IL-6受体的抗体，目前FDA也批准了托珠单抗治疗重症CRS。随着CAR-T细胞治疗在血液系统恶性肿瘤中的广泛使用，托珠单抗CRS治疗时机也需要更深入的探讨。本研究中，我们回顾性分析了托珠单抗早期干预CRS的治疗策略对B-ALL患者CRS严重程度和生存的影响。

## 病例与方法

一、病例

本研究回顾性分析了2015年10月至2021年7月期间输注 CD19 CAR-T细胞后接受托珠单抗缓解CRS反应的复发难治CD19^+^ B-ALL患者。所有患者都接受氟达拉滨（25 mg·m^−2^·d^−1^，−5～−3 d）和环磷酰胺（300 mg·m^−2^·d^−1^，−5～−3 d）清除淋巴细胞预处理。第0天开始，通过静脉输注总剂量为（0.25～1.0）×10^6^/kg的CD19 CAR-T细胞，分2～3 d输注完成。本研究中使用的CAR结构包括抗鼠CD19单链可变片段、4-1BB共刺激信号传导结构域和CD3ζ T细胞激活域。本研究得到了同济大学附属同济医院机构审查委员会的批准，所有患者均签署了知情同意书。

二、CRS治疗方案

患者输注CAR-T细胞后发生的CRS和ICANS分级依据NCCN指南[4]。托珠单抗的初次使用剂量为4～8 mg/kg，8 h后症状无缓解可再次重复使用托珠单抗治疗。根据托珠单抗干预的时机，将患者分为常规组和早期干预组。常规组患者在发生3级CRS时接受托珠单抗治疗；早期干预组患者持续高热4 h即接受托珠单抗治疗。

三、随访

输注CAR-T细胞后，前1个月每7 d、后每1个月、3个月后每3个月进行随访，随访指标包括铁蛋白，C反应蛋白，血常规，LDH，肝功能，肾功能，凝血功能和IL-6、IL-8、IL-10、TNF等血清细胞因子。患者在CAR-T细胞输注后21～28 d行骨髓细胞学检查及微小残留病（MRD）检测，并在随后的随访点再次行骨髓穿刺检查。患者行异基因造血干细胞移植或者疾病复发即为出组。总生存（OS）时间定义为从第1天输注CAR-T细胞至患者死亡或随访终点的时间。无事件生存（EFS）时间定义为从第1天输注CAR-T细胞至患者疾病进展、复发、死亡或随访终点的时间。

## 结果

一、基线临床特征

22例复发难治B-ALL患者输注CAR-T细胞发生了CRS并接受了托珠单抗治疗，男13例，女9例，中位年龄为24（9～68）岁。其中9例（40.91％）为常规组，13例（59.09％）为早期干预组。1例常规组患者既往接受异基因造血干细胞移植治疗。患者的临床特征详见表1。

**表1:** 22例复发难治急性B淋巴细胞白血病患者CAR-T细胞治疗后接受托珠单抗治疗CRS临床特征［例（％）］

临床特征	总体（22例）	常规组（9例）	早期干预组（13例）
性别			
男	13（59.19）	6（27.27）	7（31.81）
女	9（40.91）	3（13.64）	6（27.27）
年龄			
≤24岁	11（50.00）	3（13.64）	8（36.36）
>24岁	11（50.00）	6（27.27）	5（22.73）
骨髓原始细胞比例			
<20%	2（9.09）	1（4.55）	1（4.55）
≥20%	20（90.91）	8（36.36）	12（54.55）
CAR-T细胞输注剂量			
<1×10^6^/kg	5（22.73）	3（13.64）	2（9.09）
≥1×10^6^/kg	17（77.27）	6（27.27）	11（50.00）
既往接受HSCT	1（4.55）	1（4.55）	0
初次托珠单抗剂量			
4 mg/kg	4（18.18）	3（13.64）	1（4.55）
8 mg/kg	18（81.82）	6（27.27）	12（54.55）
2次托珠单抗	4（18.18）	3（13.64）	1（4.55）
托珠单抗总剂量			
4 mg/kg	3（13.64）	2（9.09）	1（4.55）
8 mg/kg	16（72.73）	5（22.73）	11（50.00）
12或16 mg/kg	3（13.64）	2（9.09）	1（4.55）

注 CAR-T细胞：嵌合抗原受体T细胞；CRS：细胞因子释放综合征；HSCT：造血干细胞移植

二、早期干预对严重CRS和ICANS的影响

常规组中，2例患者发生5级CRS（即因CRS死亡），7例患者发生3～4级CRS，其中3例接受了无创呼吸机辅助通气，3例发生肾功能损伤。早期干预组没有患者因CRS发生死亡，7例（53.85％）发生3～4级CRS，其中2例接受了无创呼吸机辅助通气，2例出现了肾功能损伤，6例（46.25％）发生1～2级CRS。无论是常规组还是早期干预组都没有患者接受血液透析治疗。4例患者接受了第二剂托珠单抗治疗，其中常规组3例，早期干预组1例，没有患者接受3次以上托珠单抗治疗。

16例（72.73％）患者发生ICANS，其中5例在托珠单抗使用前即发生了ICANS。托珠单抗使用后，常规组中5例（55.56％）发生ICANS，3例为3～4级ICANS，2例为1～2级ICANS；早期干预组中8例（61.54％）患者发生了ICANS，3～4级和1～2级ICANS分别为3例和5例。托珠单抗使用后发生ICANS的中位时间为4（1～11）d。

三、早期干预对疾病缓解和生存的影响

患者在CAR-T细胞输注后21～28 d行骨髓穿刺检查，评估治疗效果。常规组7例可评估疗效患者中5例（71.43％）获得完全缓解；早期干预组13例可评估病例中11例（84.62％）获得完全缓解。所有获得完全缓解的患者均为MRD阴性。中位随访9（2～40）个月，常规组和早期干预组患者的中位OS时间分别为5（95％ *CI* 0～11）个月、10（95％*CI* 5～15）个月；中位EFS时间分别为2（95％*CI* 0～5）个月、7（95％*CI* 3～11）个月，生存曲线见图1。

**图1:**
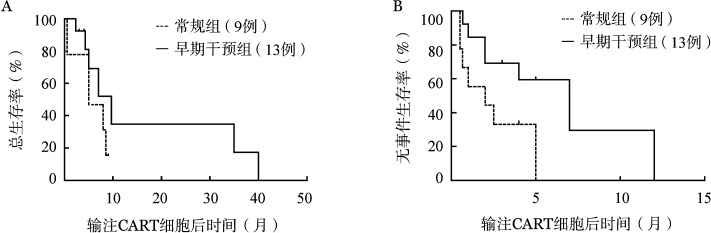
常规组和早期干预组患者嵌合抗原受体T细胞（CAR-T细胞）治疗后的总生存（A）与无事件生存（B）曲线

## 讨论

CAR-T细胞治疗复发难治B-ALL获得了65％～90％的完全缓解率[4]–[8]。然而，治疗相关毒性如CRS发生率高，需要重症监护和复杂的支持治疗限制了CAR-T细胞治疗B-ALL的广泛应用。不同的临床试验报道了CAR-T细胞治疗ALL患者重症CRS发生率从8.3％到43％不等[5]–[6],[9]–[10]。托珠单抗被FDA批准用于缓解CAR-T细胞治疗过程中重症或者威胁生命的CRS。越来越多的文献证实托珠单抗早期干预CRS不会影响CAR-T细胞治疗效果，因此托珠单抗干预CRS的时机不断提前。NCCN指南推荐持续发热3 d以上或者伴有明显合并症的1级CRS，伴有1级CRS的老年患者，2～4级CRS患者使用托珠单抗治疗CRS。目前尚无早期应用托珠单抗对CRS的严重程度以及CAR-T细胞疗效的分析。本研究将持续高热4 h确定为缓解CRS的干预时机，将22例ALL患者分为托珠单抗早期干预组和常规组，回顾性分析了托珠单抗早期干预CRS的治疗策略对ALL患者CRS严重程度、CAR-T细胞治疗效果和患者生存的影响。

既往已有类似临床研究发现早期应用托珠单抗可降低ALL患者CAR-T细胞治疗重症CRS的发生率。Gardner等[11]将静脉补液可纠正的低血压，或持续发热10 h以上或轻度低氧血症定义为轻度CRS，对20例持续存在轻度CRS患者采取托珠单抗联合糖皮质激素的早期干预策略；剂量限制性毒性（DLT）组患者在出现持续48 h以上的4级CRS反应后接受托珠单抗联合或不联合糖皮质激素的治疗，两组的CRS发生率相似，但进一步分析发现DLT组重症CRS的发生率为30％，早期干预组仅为15％。Kadauke等[12]的多中心前瞻性研究中按照骨髓原始细胞≥40％和<40％分为高肿瘤负荷组和低肿瘤负荷组，高肿瘤负荷组患者如24 h内2次体温≥38.5 °C（至少间隔4 h）采取托珠单抗的早期干预治疗策略，低肿瘤负荷组患者接受常规CRS治疗。研究发现，高肿瘤负荷组4级CRS的发生率为20％，而低肿瘤负荷组4级CRS的发生率为3.6％，对于高肿瘤负荷患者提前使用托珠单抗治疗降低了预期的4级CRS发生率。本研究再次提前了托珠单抗早期干预的时机，早期干预组患者输注CAR-T细胞后持续高热4 h即接受托珠单抗治疗。常规治疗组中所有患者都发生了3级及3级以上的CRS，2例患者发生了5级CRS；早期干预组中有 7例（53.85％）患者发生了3～4级CRS，没有患者发生5级CRS。因此本研究提示托珠单抗早期干预可能发生的重症CRS可以降低患者在CAR-T细胞治疗过程中的死亡率。

Gardner等[11]分析了托珠单抗联合或不联合激素早期干预CAR-T细胞治疗ALL患者产生的CRS反应，早期干预组和DLT组患者MRD阴性的完全缓解率分别为91％和95％，CRS的干预措施不仅没有影响患者的缓解率也没有影响缓解的持续时间。此外，随着CAR-T细胞治疗范围扩大到B细胞淋巴瘤和多发性骨髓瘤，托珠单抗的应用除了不影响CAR-T细胞对ALL的治疗效果，也不影响CAR-T细胞治疗大B细胞淋巴瘤和多发性骨髓瘤的有效性。100例可评估的大B细胞淋巴瘤患者接受了抗CD19 CAR-T细胞治疗后，中位随访10个月后发现糖皮质激素的累积剂量越高无进展生存时间越短，而是否使用托珠单抗与患者的无进展生存无关[13]。CAR-T细胞治疗多发性骨髓瘤的研究中，在发热后12 h内使用托珠单抗的早期治疗策略同样没有发现增加治疗相关的毒性或者降低治疗效果[14]。本研究中的早期干预组和常规组患者的完全缓解率分别为 84.62％和71.43％，并且所有取得完全缓解的患者均为MRD阴性的完全缓解，提前使用托珠单抗没有降低本研究中患者的完全缓解率。

ICANS的危险因素为老年、既往有神经系统疾病、疾病负荷高、CAR-T剂量、预处理中使用氟达拉滨和正在发生的重症CRS[15]–[18]。ZUMA-3[19]研究入组了71例难治复发的成人ALL患者，输注CAR-T细胞后神经系统事件的发生率为60％，3级及以上的事件发生率为25％。Gu等[20]采用新结构的 CD19 CAR-T（HI19α-4-1BB-ζ CAR T）治疗难治复发ALL患者，65％的患者发生了ICANS，3级及以上ICANS占40％。Qi等[21]对40例伴有中枢神经系统白血病的患者进行了CAR-T细胞治疗，11例（22.9％）患者发生了3～4级神经毒性事件。本研究中CAR-T细胞治疗后6例（27.27％）患者出现了3～4级ICANS，早期干预组中3例（23.08％）发生了3～4级ICANS，早期使用托珠单抗同样并未增加ICANS的发生。

综上所述，早期使用托珠单抗干预CRS有助于减少5级CRS反应的发生，为CAR-T细胞治疗ALL所产生的CRS反应的处理提供了更优化的治疗策略。
